# Long live the queen, the king and the commoner? Transcript expression differences between old and young in the termite *Cryptotermes secundus*

**DOI:** 10.1371/journal.pone.0210371

**Published:** 2019-02-13

**Authors:** José Manuel Monroy Kuhn, Karen Meusemann, Judith Korb

**Affiliations:** Evolutionary Biology and Ecology, Albert-Ludwigs-Universität Freiburg, Freiburg, Baden-Württemberg, Germany; Arizona State University, UNITED STATES

## Abstract

Social insects provide promising new avenues for aging research. Within a colony, individuals that share the same genetic background can differ in lifespan by up to two orders of magnitude. Reproducing queens (and in termites also kings) can live for more than 20 years, extraordinary lifespans for insects. We studied aging in a termite species, *Cryptotermes secundus*, which lives in less socially complex societies with a few hundred colony members. Reproductives develop from workers which are totipotent immatures. Comparing transcriptomes of young and old individuals, we found evidence for aging in reproductives that was especially associated with DNA and protein damage and the activity of transposable elements. By contrast, workers seemed to be better protected against aging. Thus our results differed from those obtained for social insects that live in more complex societies. Yet, they are in agreement with lifespan estimates for the study species. Our data are also in line with expectations from evolutionary theory. For individuals that are able to reproduce, it predicts that aging should only start after reaching maturity. As *C*. *secundus* workers are immatures with full reproductive options we expect them to invest into anti-aging processes. Our study illustrates that the degree of aging can differ between social insects and that it may be associated with caste-specific opportunities for reproduction.

## Introduction

“Mors certa–hora incerta” (Anselm of Canterbury): all living organisms die at some point due to extrinsic factors (extrinsic mortality) and/or intrinsic aging processes (intrinsic mortality, senescence). Aging is associated with time passing by and for most living organisms with a progressive deterioration of the body, which becomes apparent by a decline in motor and cognitive performance, a collapse of physiological processes and a decline in the ability to deal with various sources of stress [1–3]. Ultimately, evolutionary theory explains aging by a decline of the force of natural selection with age [4–7] and for individuals that are able to reproduce it predicts aging to start only after the onset of maturity [8]. At the proximate level, multiple studies have investigated the molecular mechanisms underlying aging and they identified several gene pathways, including the insulin/insulin-like growth factor (IGF) signaling (IIS) pathway [9,10] and the target of rapamycin (TOR) signaling pathway [11,12]. Other important mechanistic reasons that have been linked to aging are oxidative stress and DNA damage [13,14], chromatin instability and transposable elements (TEs) [15–18], and telomere attrition and shortening [19].

**Fig 1:**
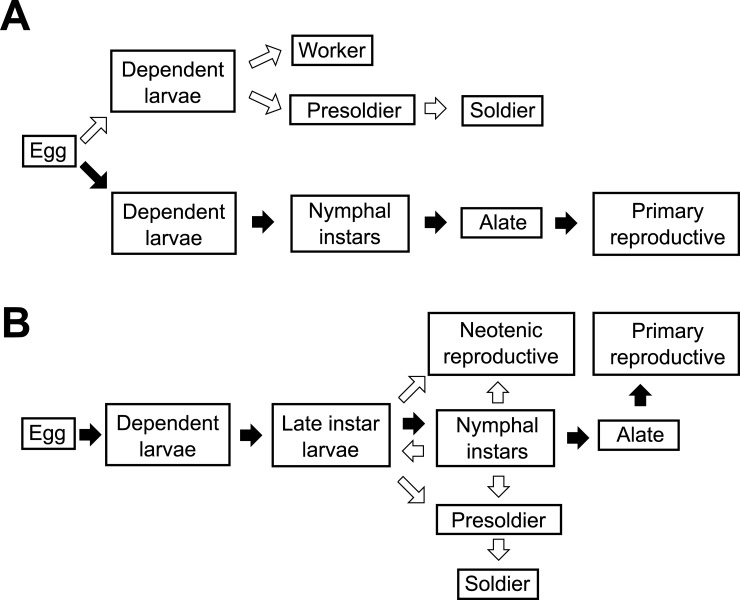
Developmental pathways in termites. Developmental pathways in termites with different degrees of sociality. (A) Typical developmental pathway for a termite with high degree of sociality, e.g. *Macrotermes bellicosus* (foraging termites). The development is less flexible and the immature workers are not totipotent. Early on during development (here in the egg stage) caste fate is determined. Individuals can either develop along the apterous line into workers and soldiers or along the nymphal line into primary reproductives. Arrows indicate the transition of one stage to another; in white: the default nymphal line which occurs in all insects, in black: deviations from these trajectories, specific to termites. (B) Typical developmental pathway for a termite with low degree of sociality, e.g. *Cryptotermes secundus* (wood-dwelling termites). Termite workers are totipotent immatures that can develop into soldiers or two kinds of reproductives, primary or neotenic reproductives. They also can molt regressively (arrow pointing backwards).

Despite aging being widespread among taxa there are huge variations in the aging rate between species. The queens of social insects (and in termites also the kings), such as honeybees, ants, termites and some wasps, not only have extraordinarily long lifespans for insects, but also can reproduce for decades [20–22]. By contrast, the non-reproducing workers are generally shorter-lived (few months up to several years), despite sharing the same genetic background. Outside social insects such differences in lifespan can only be found between species. This makes social insects promising new models for aging research. The ultimate reasons why social insect reproductives can live so long are still rarely addressed (reviewed in [23,24]). The worker’s reproductive capacity is expected to play a major role in explaining the difference in longevity between queens and workers. In species with sterile workers, the queen to worker longevity ratio should be larger than when workers can reproduce. When workers have full reproductive options–as is the case for some termites–they resemble non-social organisms in that aging should start only after reaching maturity. This is not the case for sterile workers. In addition, if workers can inherit the breeding position of a colony, we expect a reduced selection for longevity in queens. Studies on the proximate mechanisms that can help in testing such hypotheses are starting to accumulate, although they are still concentrated on the honeybee *Apis mellifera*, the bumblebee, *Bombus terrestris*, and a few ant species (e.g., reviews for honey bee: [25,26]; ant species [27–30]; bumblebee: [31]). Only three studies exist that address polyneopteran social insects, the termites, [18,32,33] which evolved eusociality independently from social Hymenoptera (wasps, bees and ants). These termite studies have in common that they address species with clearly morphologically distinct castes, where dispersing reproductives and workers do not share the same developmental pathway (Fig 1A) and workers have only reduced or no reproductive options. In order to understand how the queen: worker difference in longevity evolved with sociality, it may help to investigate socially less complex species in which workers have full reproductive options. This is our aim in the current study.

Assuming that gene expression differences reflect phenotypic differences at the protein- and physiological level, we analyzed transcript expression data of young and old workers, queens and kings of the drywood termite *Cryptotermes secundus* (Hill, 1925) (Blattodea, Isoptera, Kalotermitidae). *C*. *secundus* workers are totipotent immatures and all reproductives develop from these workers, there is no distinction between a reproductive and a neuter line (Fig 1B) [34–36]. This linear developmental trajectory occurs in all wood-dwelling termite species (also called one-piece nester, [37]), including all Kalotermitidae and Archotermopsidae and a few Rhinotermitidae [34,35], and it is widely accepted as ancestral in termites [36]. There are two types of reproductives: primary reproductives that found new colonies after a nuptial flight as alates, and neotenic secondary reproductives that inherit the colony after the death of the current reproductives. The colony size in *C*. *secundus* is rather small (generally around 300–400 individuals) [38]. Reproductives of *C*. *secundus* can live up to 10–13 years in the laboratory [22]. Yet the median maximum lifespan of *C*. *secundus* is only 6–7 years, even under optimal laboratory conditions, while that of *M*. *bellicosus* is around 10–11 years under high extrinsic mortality inherent to the field (e.g. predation) [18]. The longevity of the workers from both species is reversed; the sterile *M*. *bellicosus* workers live a few months only [18], whereas the totipotent *C*. *secundus* workers are rather long-lived as they need a minimum of four to five years to become reproductives [22]. We analyzed transcript expression differences between young and old workers, and young and old kings and queens of *C*. *secundus*. We compared our results with those obtained for the solitary model insect *Drosophila melanogaster* [39–42] and two social insects which live in more complex societies, the ant *Cardiocondyla obscurior* [43] and the termite *M*. *bellicosus* [18]. Our study contributes novel insights into how aging changes with sociality.

## Materials and methods

### Sample collection, preparation, sequencing and library construction

For this study, *C*. *secundus* colonies were collected near Palmerston- Channel Island from a mangrove ecosystem (12°30’ S, 131°00’ E; Northern Territory, Australia) [38]. The colonies were transferred to wooden blocks of *Pinus radiata*. After transport to the laboratory, the stock colonies were kept under standardized conditions in a climate room with a temperature of 28°C, 70% humidity and a 12h day/night rhythm. Under these conditions colonies develop as in the field (see [34]).

Total RNA was extracted from twelve different individuals belonging to nine different colonies: four primary queens, four primary kings and four workers (two young and two old individuals of each caste, S1 Table). Young queens and kings were collected one year after the nuptial flight (yearling reproductives). The age was determined by the color of the cuticle, tunnel size, and colony size, which was approximately 20 workers and one soldier. The old primary reproductives had been reproducing for at least seven years. Young workers were selected from the youngest worker instars, no more than six months old. The age of the old workers was at least three years, based on the instar (last larval worker instar) and the minimum developmental time it takes to reach this instar [34]. They might be older due to further stationary molts (size remains the same and no morphological changes take place) or regressive molts (decrease in size and wing-bud development). The sex of *C*. *secundus* workers can currently not be determined; there are no reliable morphological differences between male and female workers and attempts with traditional karyological methods failed.

An in-house protocol was followed for RNA extraction. Briefly, individuals were placed on ice and the gut was removed and discarded. Whole bodies were then used for RNA extraction. Samples were transferred into peqGOLD Tri Fast (PEQLAB) and homogenized in a Tissue Lyser II (QIAGEN). Chloroform was used for protein precipitation. From the aqueous phase, RNA was precipitated using Ambion isopropyl alcohol and then washed with 75% ethanol. Obtained pellets were solved in nuclease-free water. DNA was subsequently digested using the DNase I Amplification Grade kit (Sigma Aldrich, Cat. No. AMPD1). We performed an RNA Integrity Number Analysis (RIN Analysis) measuring the RNA concentration with the Agilent RNA 6000 Nano Kit using an Agilent 2100 Bioanalyzer (Agilent Technologies) for quality control. Samples with total RNA were sent on dry ice to BGI Tech Solutions (HONGKONG) Co. and then to Shenzhen, PR China for sequencing. The preparation of the cDNA libraries was performed by BGI according to their internal and proprietary standard operating procedure. The company performed paired-end sequencing (not-strand specific) on Illumina HiSeq platforms (S1 Table).

### RNA-seq data analysis

The raw reads’ quality was evaluated with FastQC (v0.11.4, [44]). Common Illumina- and BGI in-house adapter sequences were trimmed from the raw reads using Trimmomatic (v0.33, [45]) and we kept only reads with a minimum length of 70 bp (for a rationale see [46]). Expression differences were assessed at the level of the transcripts. Reads were pseudo-aligned with Kallisto (v0.43.0, [47]) against *C*. *secundus* transcriptome obtained from a draft version of the *C*. *secundus* genome (provided as supplementary material on Dryad) [48]. The counts estimated by Kallisto were rounded to the nearest integer and used to check the completeness of our samples with BUSCO (v. 3.0.2 [49]).The protein coding sequences of the transcripts that had at least one positive match (n>0 in count table) were used as input for BUSCO. We ran BUSCO (at the protein level -m prot) against the insect gene set of ortholog groups (insecta odb 9) with default settings. This gene set includes 1,658 single copy orthologs.

The Kallisto counts were used as input for DESeq2 (v1.10.1, [50]) in R (v3.2.3, R Core Team 2015) to determine transcript expression differences between old and young individuals of queens, kings and workers. In DESeq2 the p-values are adjusted for multiple testing using the false discovery rate (FDR) approach [51]. In order to correct for unaccounted sources of variation the ‘Surrogate Variable Analysis’ (sva) package (v 3.20.0) as implemented in R was used [52,53]. The software identifies and estimates surrogate variables for unknown sources of variation (for instance, batch or colony effects). For data visualization, a principal component analysis (PCA) was performed with DESeq2 using transformed count data (variance stabilization). The age-specific differentially expressed transcripts (DETs) were compared between castes and the overlaps were visually represented with Venn diagrams generated using the online tool Venny (v2.1 [54]) and graphically processed with Inkscape (v0.91, www.inkscape.org).

### Functional annotation and enrichment

A draft version of the *C*. *secundus* genome was used (provided as supplementary material on Dryad) [48] to obtain nucleotide and protein sequences. DETs were annotated via a BLASTX search (CBI BLAST suite v. 2.3.0, [55]) against the protein coding sequences of the termite *Zootermopsis nevadensis* (official gene set version 2.2, [56]) with a threshold E-value of 1e-05. The annotation was complemented by searching DETs at the amino acid level against the InterPro database with the software InterProScan (v5.17–56.0, [57]), using default settings. We also searched the translated sequences against the Pfam database (Pfam A, release 30) [58] with the software *hmmscan* (option—cut_ga, HMMer v3.1b2, [59]). Additionally, we looked for possible transposable element (TE) sequences by searching against the Dfam database (v2.0, [60]) using nhmmer [61] with default settings. To further assist the annotation, we inferred a set of clusters of orthologous groups (COGs) from the official gene sets at the amino-acid level of four species with (draft) genomes: *C*. *secundus* draft version, the termites *Macrotermes natalensis* (official gene set version 1.2 [62]) and *Z*. *nevadensis* (official gene set version 2.2, [56]), and the fruit fly *D*. *melanogaster* (version r6.11, [63]). For all species, we only kept the longest isoform per gene and additionally removed sequences with internal stop codons and/or selenocysteines (U). The latter causes problems in downstream analyses and sometimes sequences with selenocysteine are considered to be potential pseudo-genes (see [48]). COGs were inferred among the four reference species using the software OrthoFinder (v. 0.4.0 [64]).

A functional enrichment analysis (GO enrichment) was done with DAVID (v6.8, [65]). For this we made a second set of COGs (see above), but only with *C*. *secundus* and *D*. *melanogaster*, and additionally a BLASTP search of *C*. *secundus* sequences against the protein coding sequences (longest isoforms only) of *D*. *melanogaster* with a threshold E-value of 1e-05. The set of homologs used for the GO enrichment consisted of (i) single copy 1:1 orthologs and (ii) homologs (not single copy 1:1 orthologs) found via the BLASTP search and filtered using the best bit scores.

### Gene identification

We identified COGs for genes of interest (see S2 Table for genes of interest) and retrieved all protein coding sequences of the respective COG from OrthoDB v. 9.1 [66] for the following species: *D*. *melanogaster* (DMEL), *Apis mellifera* (AMEL), *Cardiocondyla obscurior* (COBSC), *Pollistes dominula* (PDOM), *Tribolium castaneum* (TCAS), *Z*. *nevadensis* (ZNEV) and *Blattella germanica* (BGER) (sequences provided on Dryad for each COG). COGs were found using the text search option available in OrthoDB (search for gene name or IDs e.g. “AGO2”, “FBgn0087035”). We aligned the sequences separately for each COG with MAFFT (v7.294b), with the G-INS-i algorithm default settings [67]. We built a profile hidden Markov model (HMM) from each multiple sequence alignment using hmmbuild (HMMER v3.1b2). We then used the HMM to search with hmmsearch against *C*. *secundus* and *M*. *natalensis* protein coding sequences to identify candidate sequences for each COG. For inference of gene trees, we only kept sequences with a threshold E-value of ≤1e-40. All candidate sequences were searched with hmmscan against the Pfam database (Pfam A, release 30).

Phylogenetic trees of closely related genes were inferred for a total of 26 genes of interest provided on Dryad. We included sequences of all species mentioned above that passed the threshold (≤1e-40). In all sequences, selenocysteine (U) was replaced by “X“. Sequences were aligned as described above. We subsequently identified ambiguously aligned sequence regions with Aliscore (v. 2, [68,69]) (settings: -r: maximal number of pairwise comparisons) and subsequently removed those sections with Alicut (v. 2.3, https://www.zfmk.de/en/research/research-centres-and-groups/utilities, masked alignments provided on Dryad). We used IQ-Tree (v. 1.5.5, [70]) to infer phylogenetic gene trees (single genes or groups of closely related genes) using the Maximum-Likelihood approach separately for genes (gene groups) of interest. Using parsimony start trees, we estimated the best model with the implemented ModelFinder [71] for available standard models, including the free rate models LG4M and LG4X [72]. We used default settings for rates, number of rate categories, and the Bayesian Information Criterion (BIC) to estimate the best substitution model. Statistical support was inferred from 2,000 non-parametric bootstrap replicates. We visualized the unrooted trees with bootstrap support using Seaview (v4.5.4, [73]) and graphically processed the trees with Inskape (v0.91, www.inkscape.org) (provided in Dryad).

All raw sequencing reads are deposited on EMBL (Primary Accession PRJEB27153, for sample accession numbers see S1 Table). Additional supplementary data can be accessed from the Dryad repository (dryad link can be provided upon acceptance).

### Comparison with other species

The results obtained in this study were compared with similar aging studies for the fruit fly *D*. *melanogaster* [39–42,74], the ant species *C*. *obscurior* [43], and the termite *M*. *bellicosus* [18], the only termite of which comparable aging data exist. Comparisons were done at three different levels: (i) differentially expressed genes (DEGs) between old and young individuals for the studies of Elsner et al. (2018) [18], von Wyschetzki et al. (2015) [43] and Lai et al. (2007) [39]; (ii) GO enriched terms for the studies of Elsner et al. (2018) [18], Wyschetzki et al. (2015) [43], Doroszuk et al. (2012) [41] and Pletscher et al. (2002) [74]; and (iii) candidate markers of aging and lifespan regulation for the studies of Doroszuk et al. (2012) [41], Lai et al. (2007) [39], Magwire et al. (2010) [40] and Carnes et al. (2015) [42]. For *C*. *obscurior* and *M*. *bellicosus* we used the corresponding annotation of *D*. *melanogaster* 1:1 orthologs and other homologs [18,43]. In addition, *C*. *secundus* DETs were compared with *D*. *melanogaster* ‘aging genes’ available in the ageing gene database (GenAge Build 18, [75]).

## Results

### Differential expression between age-classes within castes in *C*. *secundus*

**Fig 2:**
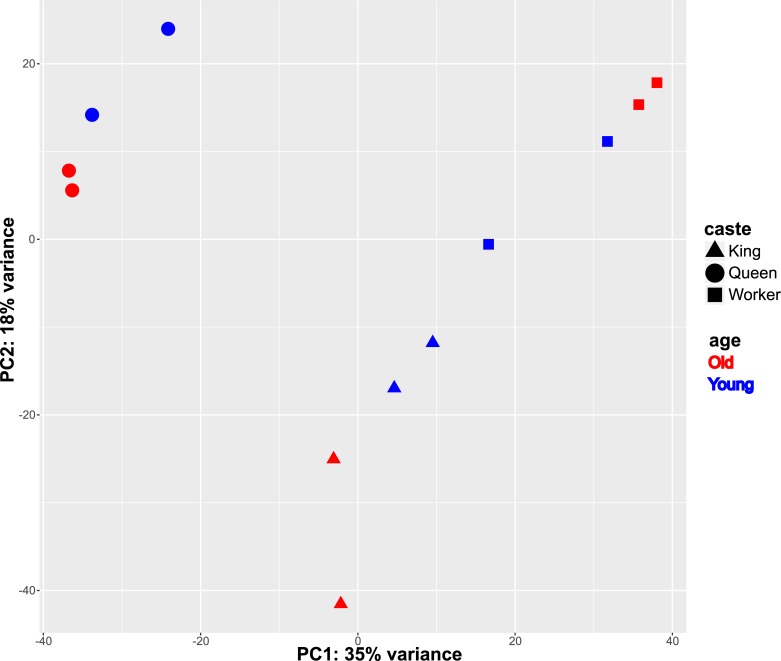
Principal component analysis of transcript count data. Principal component analysis of transcript count data with variance stabilizing transformation for two age classes of reproductives and workers.

**Fig 3:**
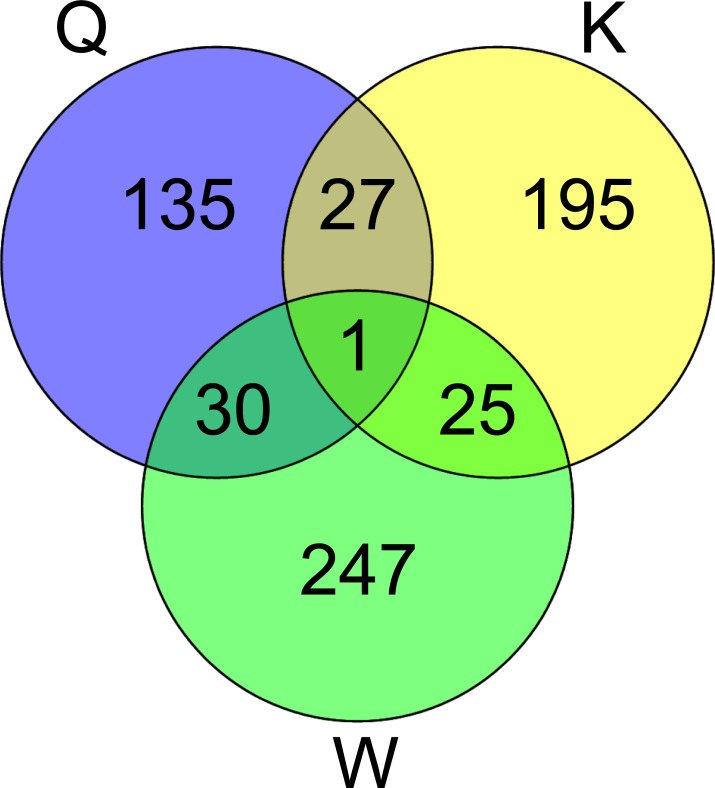
Venn diagram of differentially expressed transcripts. Shared differentially expressed transcripts (DETs) with age (old vs. young) between *C*. *secundus* queens (Q), kings (K) and workers (W). Blue: old queens vs young queens, yellow: old kings vs young kings and green: old workers vs young workers.

**Fig 4:**
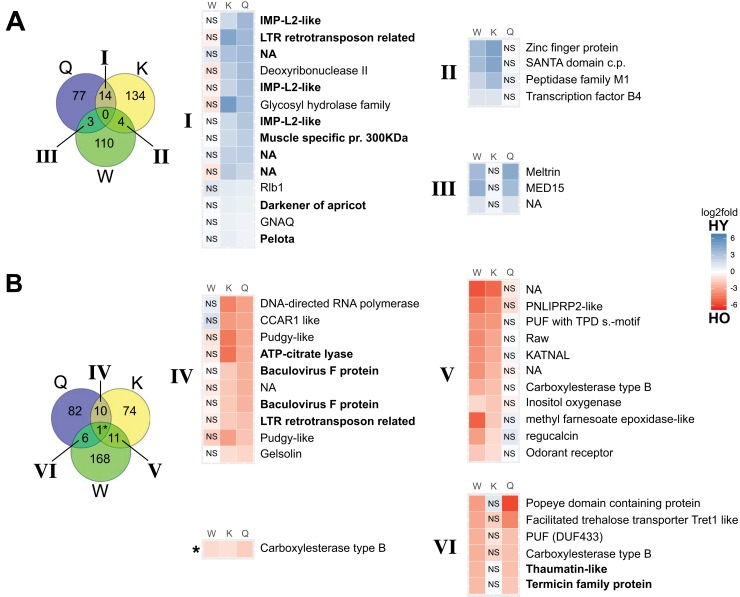
Venn diagrams and heatmaps of differentially expressed transcripts. Shared differentially expressed transcripts (DETs) with age (old vs young) between *C*. *secundus* queens (Q), kings (K) and workers (W). The heatmaps depict the log2fold changes in expression within castes, only DETs. Each row corresponds to a DET and each column to a caste. NS: differential expression not significant (p^adj^>0.05); transcripts in bold are discussed in the text. Roman numerals link the DETs shared between castes to the heatmaps. (A) DETs more highly expressed in young compared to old individuals (HY). (B) DETs more highly expressed in old compared to young individuals (HO). In Venn diagrams, blue: old queens vs young queens, yellow: old kings vs young kings and green: old workers vs young workers.

The results of the BUSCO analysis showed for each sample a completeness of more than 90% of the insect gene set of OGs (insecta odb 9) (see S1 Table). Using transcript transformed count data, the PCA separated the castes on the first component, which explained 35% of the variance (Fig 2). The queens clustered together and were clearly separated from the other castes, whereas the kings clustered closer to the workers. The second component explained 18% of the variance and separated the age classes. The age class pattern was reversed in the workers in comparison to the reproductives (Fig 2). Two surrogate variables were estimated with the package sva [53], and they were included in the formula for differential expression testing to correct for unaccounted sources of variation. A total of 815 transcripts were significantly differentially expressed (DETs) between the age-classes within castes: 193 for the queens, 248 for the kings, and 374 for the workers (padj < 0.05, S2 Table and Fig 3). These caste-specific age-related DETs were divided into two groups: transcripts that were more highly expressed in younger individuals than in older ones (HY) and transcripts that were more highly expressed in old individuals compared to young ones (HO) (Fig 4). Only one HO DET, annotated as a carboxylesterase like gene, was shared across all castes (see Fig 3); 10% of the DETs were shared in a pairwise manner. Hence, most DETs were caste specific. DETs in the workers were involved in a plethora of different functions including metabolic processes, growth, development and morphogenesis, regulation of transcription, alternative splicing and chromatin remodeling (S2 and S3 Tables).

### Differential expression of genes linked to aging

The DETs that were considered important for aging were split into the following categories: (i) DNA damage response, genome stability and telomeres; (ii) transposable elements (TEs); (iii) oxidative stress; (iv) neural aging; (v) reproduction, (vi) immunity and (vii) other age-related genes.

#### DNA damage response, genome stability and telomeres

Two DETs more highly expressed in young compared to old queens (HY) were linked to DNA damage repair, genome stability and telomere maintenance: serine/threonine-protein kinase ATR (*ATR/mei-41*) and *Ku80* [76–79]. Linked to DNA repair in kings were three HY and one HO DETs: ‘mismatch repair endonuclease PMS2’ (*Pms2*), ‘MORF related gene 15’ (*MRG15*), ‘Translationally controlled tumor protein’ (*Tctp*) [80–82] and *fancl* [83]. Other important HY DETs in kings were *kin17* protein related to DNA damage response [84], and nucleosome assembly protein1 (*nap1*) related to telomere maintenance [85]. The workers shared with the queens the expression of *ATR* but in opposite direction (HY in queens and HO in workers, S1 Fig) and had another HO DET with homology to *ATR* (not shared with the queens, S2 Table). Workers had additionally three more HY DETs involved in telomere maintenance, DNA repair and DNA damage response: regulator of telomere elongation helicase 1 (*RTEL1*), *nap1* (shared with kings HY), and DNA mismatch repair protein Mlh1-like (*Mlh1*) [80,85,86].

#### Transposable elements

Seven HY and thirteen HO DETs in queens were linked to TEs. Out of these, two HY and three HO were shared with the kings (Fig 4A and 4B). One of the shared HY DETs was identified as ‘pelota’ (*pelo*), a gene that encodes a conserved protein involved in transposon silencing [87]. Kings had in total eight HY and seven HO DETs related to TEs. One of the HY DETs in kings was identified as a homolog of *vasa*, a gene involved in transposon silencing in the germline by participating in piRNA biogenesis and amplification [88], and another one was identified as ‘Heat shock protein cognate 4’ (Hsc70-4), which is involved in the RNA interference pathway (RNAi) and in heterochromatin formation [89,90]. Strikingly, 21 DETs in workers were associated with TEs and contrary to the reproductives most of these DETs were more highly expressed in young compared to old individuals: 19 HY and two HO (S2 Table). One of the HY DETs in workers was identified as ‘eggless’, which is an H3K9 methyltransferase that interacts with heterochromatin protein 1 (HP1) to spread heterochromatin, and it is necessary for TE silencing via piRNA pathway [91,92]. Of the two HO DETs in workers, one was annotated as ‘Vacuolar H+ ATPase 16kD subunit 1’ (Vha16-1). Vha16-1 participates in the uptake of dsRNA to the cell, which allows the activation of the RNAi pathway [93].

#### Oxidative stress

In queens and kings, one HY DET was identified as darkener of apricot (*Doa*) (Fig 4A), which among other functions can negatively regulate the expression of superoxide dismutases (*SOD1* and *SOD2*) in *D*. *melanogaster* [94]. Additionally, in kings the GO enrichment revealed for the HY DETs multiple GO BP terms related to catabolism of xenobiotic compounds and insecticides (S3 Table). The signal for these BP terms originated from seven HY DETs annotated as cytochrome P450 family 6 (CYP6). Another HY DET in kings was identified as a catalase-like gene. Other DETs in kings potentially involved in oxidative stress were identified as cytochrome P450s family 4 (CYP4, one HY and two HO), cytochrome P450 of unknown family (HY, homologue to CYP303a1), and one mitochondrial uncoupling protein (HY), which can modulate and reduce ROS production [95]. In workers multiple transcripts were associated to oxidative stress: two catalase-like proteins (both HO, one of them shared in opposite direction with kings, see S1 Fig), a homolog of ‘inactivation no afterpotential E’ (HO, *inaE* in *D*. *melanogaster*)”, a carbonic anhydrase (HO), isocitrate dehydrogenase (HO) and five cytochrome P450s from different families (one HY and four HO, see S2 Table). P450 genes, however, can also play an important role in caste differentiation in termites and hence need not be linked with oxidative stress.

#### Neural aging

In queens three HY DETs were potentially linked to aging of the brain: Palmitoyl-protein thioesterase 1 (*Ppt1*) and two Down syndrome cell adhesion molecules (Dscam) [96,97]. One of the Dscam proteins was shared with workers but the change in expression with age was in opposite direction (HY in the queens but HO in the workers, S1 Fig). In kings, one HY DET was identified as a homolog of apolipoprotein D (*ApoD*). ApoD is mainly expressed in the brain and nervous system and its over expression in *D*. *melanogaster* increases lifespan and stress resistance [98,99]. Additionally in kings, the 1:1 ortholog of refractory to sigma p (ref(2)p), a marker of neuronal aging and an important component of the mitochondrial unfolded protein response [100], was upregulated (HO).

#### Reproduction

In queens four DETs were linked to fertility signaling and reproduction: *Neofem2* and Follicle cell protein 3C (*Fcp3C*, both HO), and two homologs of *D*. *melanogaster* gene CG33981 (HY) [101–103]. Both CG33981 homologs, were expressed in workers in opposite direction compared to queens (HO in workers and HY in queens, S1 Fig). Multiple GO terms connected to reproduction were enriched in the HY transcripts of kings (see S3 Table). This signal came from multiple transcripts including *pelo*, *hsc-4* and *nap1* that are involved in transposon silencing, heterochromatin formation and genome stability (see Transposable elements).

#### Immunity

Several DETs between age-classes were related to immunity. Two DETs were shared between queens and workers in the same direction (HY, Fig 4), and were identified as a ‘thaumatin-like’ protein and a ‘termicin family’ protein. These proteins are involved in immune defense against fungal infections [104–106]. Queens and kings shared the differential expression of ‘modular serine protease’, which is involved in innate immunity [107,108], but in opposite direction (HY in kings and HO in queens). In kings, two more HO DETs were linked to immunity: an antimicrobial peptide Attacin, and a protein containing a ‘single domain Von Willebrand factor type C’ (SVWC). Proteins with the SVWC domain are involved in response to bacterial infections and viral challenges (see [109]). In workers two genes involved in innate immune response coding for a transglutaminase and a peptidoglycan recognition protein [110,111] were significantly differentially expressed (both HO).

#### Other age-related genes

The 1:1 ortholog of ATP citrate lyase (ATPCL), a gene that in *D*. *melanogaster* males has a pro-aging effect [112], was upregulated in old reproductives (HO, Fig 4). In contrast to ATPCL, the 1:1 ortholog of ‘ATP synthase, subunit D’ (ATPsynD), whose downregulation in *D*. *melanogaster* with low carbohydrate to protein diet has been associated to a lifespan extension [113], was downregulated in old kings (HY).

In old workers, the 1:1 ortholog of ‘S-adenosylmethionine Synthetase’ (Sam-S) was downregulated. The decrease of S-adenosylmethionine has been linked to a lifespan extension in *C*. *elegans* and *D*. *melanogaster* [114,115].

### Comparison with other species

The results for the age-associated expression differences in *C*. *secundus* were compared with the results of similar aging studies for reproducing females of a solitary insect, *D*. *melanogaster*, and two social insects, the ant *C*. *obscurior* and the termite *M*. *bellicosus* (using the respective orthologs or homologs to *D*. *melanogaster*). We concentrate on *C*. *secundus* queens here as kings (males) were not studied in the other species. Results of the kings of *C*. *secundus* are provided in S3 and S4 Tables.

#### Comparison with D. melanogaster

Regarding GO enrichment, *C*. *secundus* queens shared only five GO terms with the flies [41], only one in the same direction (Table 1). In workers ten GO terms were shared with *D*. *melanogaster* females in opposite direction and nine GO terms were shared in the same direction. One GO term of interest was ‘oxidation-reduction process’ (oxidative theory of aging) (see Table 1). 30 DETs in *C*. *secundus* were shared with the candidate markers of aging and lifespan determination of the studies of Lai et al. (2007) [39], Doroszuk et al. (2012) [41] and Carnes et al. (2015) [42] (S6 Table). In the reproductives, four HY DETs (four transcripts in queens and two of these shared by kings, same gene) were identified as homologs of Ecdysone-inducible gene L2 (*Imp-l2*), which is a gene contained in the GenAge database.

**Table 1:** Comparison of GO enriched terms between species.

**Queens**									
**GO Term**	**GO ID**	**Csec**	**CO**	**Mb_MW**	**DM**	**# genes Csec**	**# genes CO**	**# genes Mb_MW**	**# genes DM**
striated muscle tissue development	GO:0014706	-	NA	NA	+	3(19)	NA	NA	12
anatomical structure homeostasis	GO:0060249	-	+	NA	+	5(118)	4(31)	NA	13
muscle tissue development	GO:0060537	-	NA	NA	+	3(24)	NA	NA	12
actomyosin structure organization	GO:0031032	-	-	NA	+	4(72)	5(23)	NA	12
cell development	GO:0048468	-	+	+	NA	17(1321)	19(475)	378(1138)	NA
regulation of biological quality	GO:0065008	-	NA	+	NA	14(1023)	NA	318(929)	NA
chromosome organization	GO:0051276	-	+	NA	-	8(437)	13(182)	NA	165
synapse assembly	GO:0007416	+	NA	+	NA	3(36)	NA	62(140)	NA
**Workers**									
**GO Term**	**GO ID**	**Csec**	**CO**	**Mb_MW**	**DM**	**# genes Csec**	**# genes CO**	**# genes Mb_MW**	**# genes DM**
cell adhesion	GO:0007155	-	NA	NA	+	12(365)	NA	NA	58
biological adhesion	GO:0022610	-	NA	NA	+	9(205)	NA	NA	58
DNA metabolic process	GO:0006259	-	+	NA	-	10(270)	9(159)	NA	141
organic acid biosynthetic process	GO:0016053	-	NA	NA	+	5(73)	NA	NA	25
metabolic process	GO:0008152	-	NA	-	NA	75(3998)	NA	1261(3582)	NA
actomyosin structure organization	GO:0031032	-	-	NA	+	5(72)	5(23)	NA	12
myofibril assembly	GO:0030239	-	-	NA	+	4(43)	4(11)	NA	9
epithelium development	GO:0060429	-	+	+	+	23(878)	10(166)	279(798)	46
instar larval or pupal development	GO:0002165	-	NA	+	+	17(557)	NA	190(519)	95
post-embryonic development	GO:0009791	-	NA	+	+	18(615)	NA	198(570)	100
tissue development	GO:0009888	-	NA	+	NA	25(935)	NA	291(848)	NA
synapse assembly	GO:0007416	+	NA	+	NA	10(166)	NA	62(140)	NA
oxidation-reduction process	GO:0055114	+	-	-	+	15(365)	NA	176(336)	164
protein localization	GO:0008104	+	NA	NA	-	5(44)	NA	NA	112
modulation of synaptic transmission	GO:0050804	+	NA	+	NA	7(103)	NA	41(89)	NA
morphogenesis of an epithelium	GO:0002009	+	+	+	+	3(19)	10(160)	198(524)	44
carbohydrate metabolic process	GO:0005975	+	-	NA	NA	16(363)	16(193)	NA	NA
cellular carbohydrate metabolic process	GO:0044262	+	-	NA	NA	9(193)	11(109)	NA	NA
carboxylic acid metabolic process	GO:0019752	+	-	-	NA	10(248)	19(181)	112(229)	NA
oxoacid metabolic process	GO:0043436	+	-	-	NA	10(249)	19(181)	113(230)	NA
organic acid metabolic process	GO:0006082	+	-	-	NA	11(290)	19(181)	127(268)	NA
multicellular organism reproduction	GO:0032504	+	+	+	-	5(49)	20(373)	234(745)	183
regulation of nervous system development	GO:0051960	+	NA	+	+	13(274)	NA	39(83)	21
synapse organization	GO:0050808	+	NA	+	+	12(244)	NA	82(195)	14
ion homeostasis	GO:0050801	+	NA	NA	+	6(99)	NA	NA	14
lumen formation, open tracheal system	GO:0035149	+	NA	NA	+	3(17)	NA	NA	4
learning or memory	GO:0007611	+	NA	+	+	7(142)	NA	65(120)	22
regulation of cell morphogenesis	GO:0022604	+	NA	+	+	8(180)	NA	66(152)	32
single-organism metabolic process	GO:0044710	+	NA	-	NA	43(1266)	NA	442(1172)	NA
small molecule metabolic process	GO:0044281	+	NA	-	NA	19(617)	NA	251(572)	NA
regulation of multicellular organismal dev.	GO:2000026	+	NA	+	NA	21(442)	NA	158(380)	NA
multicellular organismal process	GO:0032501	+	NA	+	NA	77(3138)	NA	780(2737)	NA
regulation of developmental process	GO:0050793	+	NA	+	NA	23(605)	NA	199(524)	NA
single-multicellular organism process	GO:0044707	+	NA	+	NA	66(2660)	NA	692(2349)	NA
regulation of anatomical structure morphogenesis	GO:0022603	+	NA	+	NA	13(255)	NA	85(224)	NA
regulation of multicellular organismal process	GO:0051239	+	NA	+	NA	22(608)	NA	197(524)	NA
single-organism process	GO:0044699	+	NA	+	NA	102(4866)	NA	1124(4284)	NA
system development	GO:0048731	+	NA	+	NA	50(1997)	NA	527(1766)	NA
cell-cell signaling	GO:0007267	+	NA	+	NA	19(554)	NA	185(501)	NA
single-organism behavior	GO:0044708	+	NA	+	NA	13(333)	NA	136(295)	NA
synaptic signaling	GO:0099536	+	NA	+	NA	11(258)	NA	105(234)	NA
anterograde trans-synaptic signaling	GO:0098916	+	NA	+	NA	11(258)	NA	105(234)	NA
chemical synaptic transmission	GO:0007268	+	NA	+	NA	11(258)	NA	105(234)	NA
trans-synaptic signaling	GO:0099537	+	NA	+	NA	11(258)	NA	105(234)	NA
multicellular organism development	GO:0007275	+	NA	+	NA	57(2423)	NA	626(2136)	NA
localization	GO:0051179	+	NA	+	NA	44(1792)	NA	467(1592)	NA
neuromuscular junction development	GO:0007528	+	NA	+	NA	8(161)	NA	60(136)	NA
regulation of biological quality	GO:0065008	+	NA	+	NA	28(1023)	NA	318(929)	NA
r. of synaptic growth at neuromuscular junction	GO:0008582	+	NA	+	NA	6(96)	NA	41(80)	NA
regulation of neuromuscular junction development	GO:1904396	+	NA	+	NA	6(98)	NA	41(82)	NA
single-organism cellular process	GO:0044763	+	NA	+	NA	89(4255)	NA	979(3753)	NA
regulation of synapse assembly	GO:0051963	+	NA	+	NA	6(101)	NA	42(85)	NA
cognition	GO:0050890	+	NA	+	NA	7(142)	NA	65(120)	NA
regulation of cell differentiation	GO:0045595	+	NA	+	NA	11(297)	NA	99(247)	NA

Comparison of GO enriched terms between species. Csec: *Cryptotermes secundus*, CO: *Cardiocondyla obscurior* [43], Mb_MW: *Macrotermes bellicosus* major workers [18] and DM: *Drosophila melanogaster* [41]. A minus (-) sign stands for higher expression in the young compared to old individuals, and a plus sign (+) stands for higher expression in the old compared to young individuals. NA: not applicable since not differentially expressed. # genes: total number of differentially expressed genes with this enriched GO term, number in brackets is the total amount of genes expressed (background) with this GO term.

## Comparison with the ant *C*. *obscurior*

Contrary to *D*. *melanogaster* [39], few DETs identified as *D*. *melanogaster 1*:*1* orthologs were shared with *C*. *obscurior* queens [43] (Table 1, S4 and S5 Tables). The workers shared, among others, the gene juvenile hormone epoxidase (1:1 ortholog to *D*. *melanogaster*) with *C*. *obscurior* queens (HO in both species).

### Comparison with the termite *M*. *bellicosus*

The GO enrichment results of *C*. *secundus* can only be compared with the enrichment results of *M*. *bellicosus* major workers as very few genes were differentially expressed between young and old *M*. *bellicosus* reproductives [18]. *C*. *secundus* queens shared three enriched GO terms with *M*. *bellicosus* major workers two in opposite direction and one in the same direction (Table 1). *C*. *secundus* workers shared with *M*. *bellicosus* major workers ten GO terms in opposite direction and 32 in the same direction (Table 1).Most of the GO terms were related to development, morphogenesis and metabolism.

The comparison using *C*. *secundus* DETs (without considering GO terms) was done against *M*. *bellicosus* DE genes (old vs young) of queens, kings, minor workers and major workers. Elsner et al. (2018) [18] found only two DE genes in *M*. *bellicosus* kings, and no overlap was found with *C*. *secundus* DETs. *C*. *secundus* queens shared a 1:1 orthologue with *M*. *bellicosus* queens, a cytochrome b5 (HO) (S4 Table). Additionally, the queens shared 17 genes with *M*. *bellicosus* major workers (1:1 orthologs to *Drosophila* in both species, S4 Table). Five genes showed an opposite expression pattern (HY in *C*. *secundus* queens and HO in *M*. *bellicosus* major worker) of which one was identified as an aging marker in *Drosophila*: cora [40,41] (S4 and S6 Tables). *Cora* was also upregulated in old minor workers of *M*. *bellicosus* (HO). The function of cora is not known but it interacts with forkhead box, sub-group O (*foxo*, IIS pathway) and *rictor* (mTOR pathway) [116]. Three genes were upregulated in *C*. *secundus* queens and *M*. *bellicosus* major workers (HO in both species) and nine genes were downregulated (HY in both species). Workers shared 46 genes (more than half functionally annotated) with *M*. *bellicosus* major workers, 24 in the same direction (15 HY and 9 HO) and 22 in opposite direction (all 1:1 orthologs to Drosophila in *C*. *secundus*, S4 Table).

## Discussion

We identified many age-related expression changes in *C*. *secundus* reproductives, which live in less complex colonies. This differs from the higher termite *M*. *bellicosus*, for which only very few genes were differentially expressed in the heads of young and old reproductives [18]. Our results imply that *C*. *secundus* reproductives have an earlier onset of aging than those of *M*. *bellicosus*, as supported by longevity data. *Macrotermes* reproductives can live for 20 years and more, while the maximum lifespan of *C*. *secundus* reproductives is around 10–13 years [22]. In contrast, the totipotent *C*. *secundus* workers showed fewer signs of aging in line with the hypothesis that in organisms with full reproductive options, aging is expected to start only after reaching maturity. For the present study we assumed that transcript expression differences reflected changes at the protein level (as most transcriptome studies do). This is known to be the case for certain proteins in *D*. *melanogaster* (e.g., heat shock proteins and prophenoloxidase) [117] and in bees (e.g. vitellogenin [118]), but results need to be taken with caution because transcript and protein abundances do not necessarily correspond [119–121].

### Aging in *C*. *secundus* compared to other social insect species

Age-associated expression changes in *C*. *secundus* can be related to senescence processes, such as TE-activity and its resulting DNA and protein damage. In queens and kings, DETs related to DNA damage repair and genome stability were downregulated in old compared to young individuals (S2 Table). This also applied to old workers but in addition they had two *ATR* homologs upregulated in old individuals (S2 Table). *ATR* coordinates DNA damage responses (repair and cell cycle checkpoint signaling), participates in telomere maintenance and contributes to genome stability [76–78]. This implies that the protection against damage decreased with age in the reproductives but less so in workers.

This view is also supported by our results for TE activity. TEs have been associated with aging in a broad range of animals, from *C*. *elegans*, *D*. *melanogaster*, and mice to humans [17,122–126] and most recently in termites [18]. Old queens had more TE-related transcripts than young queens and associated with this was a downregulation of a 1:1 ortholog (*D*. *melanogaster*) encoding *pelo*, which is involved in TE silencing [87]. In kings the number of TE related transcripts was similar in old compared to young kings (see S2 Table) but three genes involved in TE silencing were downregulated with age (HY): *pelo* and the homologs of *vasa* and *Hsc70-4* [87–90]. The reverse was found for workers in which the very low TE activity (two DETs) in old workers was accompanied with an upregulation of Vha16-1, which is necessary for the activation of the RNAi pathway and a systemic response against TEs [93]. In line with TE activity, was the downregulation of ATPCL (Acetyl-CoA metabolism) in the reproductives (HY). This gene has been connected to a disregulation of histone acetylation levels and the disruption of heterochromatin formation (high TE content) [112,127].

The TE-related transcripts upregulated in young workers might not be related to aging but developmental processes. Some upregulated TE-related transcripts in young workers were LINE (long interspersed nuclear elements) retrotransposons (Dfam results, S7 Table). LINE-1 retrotransposons have been shown to regulate global chromatin accessibility in the early embryo in mice [128]. Though it still requires further studies, we propose that the TE related activity in young *C*. *secundus* workers reflects regulation of complex postembryonic development of these immature instars, in line with other development-related worker DETs and the GO enrichment results (S2 and S3 Tables).

Despite the differences in tissue specificity (*C*. *secundus*: whole body without gut vs *M*. *bellicosus*: head only), which might increase the number of false negatives (see [129]), our current results compared with those for the termite *M*. *bellicosus* [18] reveal some striking similarities and differences. While the same aging-related mechanisms were detected (TE-activity and piRNA mediated TE defense), the aging pattern seems reversed between castes. Old major *M*. *bellicosus* workers were characterized by a high TE activity whereas reproductives in this ‘higher’ termite seem to be protected against TEs by the piRNA pathway [18]. This difference can be explained by the fact that the major workers of *M*. *bellicosus* are sterile, while *C*. *secundus* workers are totipotent immatures from which all reproductives derive (Fig 1B). Evolutionary theory predicts that in organisms with full reproductive options aging starts only after the onset of maturity [8]. Hence, we expect that the totipotent *C*. *secundus* workers are selected to invest in anti-aging processes, while this is less the case for sterile *M*. *bellicosus* workers in which all reproduction is channeled through a separate sexual line (Fig 1A).

Many other factors differ between the two termite species, most importantly lifestyle and associated with it, colony size and pathogen load. *C*. *secundus* is a drywood termite that nests in a single, non-decomposed piece of wood which the workers never leave to forage outside. Hence, like other drywood termites [130], *C*. *secundus* probably has a low pathogen load for workers and reproductives, and it has small colony sizes and workers (and reproductives) that experience low extrinsic mortality risks [131]. By contrast, *M*. *bellicosus* belongs to the foraging termites in which workers leave the nest to collect food and bring it back. Associated with this lifestyle are–besides reduced reproductive options for workers–larger colony sizes, and increased pathogen loads and extrinsic mortality risks, especially for workers [22]. Extrinsic mortality risks are the most important factors to influence aging in non-social organisms where all individuals can reproduce [4,8]. Hence, we also would predict faster aging of workers due to increased extrinsic mortality risks in *M*. *bellicosus*. However, these models do not consider social organisms and how things change with sterility. Models including all these factors are warranted to determine the separate contribution of different factors (e.g. worker sterility, colony size, and extrinsic mortality) for the evolution of lifespans in social insects. In termites, these factors form ‘syndromes’ associated with life type. Foraging termite species are always socially more complex, and have workers with reduced reproductive capacities and shorter lifespans than wood-dwelling species [22]. Hence, these traits seem to co-evolve and it will be difficult to separate and test the contribution of single factors.

An upregulation of genes, such as catalases and SODs, involved in the protection against oxidative stress has been associated with the long lifespan of queens of the termite *Reticulitermes speratus* in studies that compared neotenic queens and workers of unknown age [32,33]. This species lives in colonies with a degree of social complexity and worker reproductive options intermediate between *M*. *bellicosus* and *C*. *secundus*. Its developmental system is complicated and lifespan data have not been published. As a foraging species, it has bifurcated development, hence workers have reduced reproductive options but they seem to be not fully sterile. Similar to *C*. *secundus*, founding queens (primary queens) can be replaced in *R*. *speratus*. However, they seem to be rather short lived and replacements are produced from nymphal instars via parthenogenesis [132]. We also found evidence that oxidative stress plays a role in aging in *C*. *secundus*, but it supports the view that non-sterile workers, in contrast to the reproductives, increase their protection with age: catalase (two DETs), inaE, carbonic anhydrase and idh—all genes important in oxidative stress defense [133–136]—were upregulated in old compared to young workers. The expression of one catalase (upregulated in old workers) was downregulated in old kings and the 1:1 ortholog of *doa* was downregulated in old reproductives. *Doa* negatively regulates the expression of *SOD1* and *SOD2* [94]. Although cytochrome P450s from the families CYP6 and CYP4 were differentially expressed in workers, these genes might be connected to caste differentiation rather than oxidative stress [137,138], and for kings, other functions for the differentially expressed CYP4 can also not be excluded.

### Neural aging

Neurodegeneration and the decline in cognitive functions is characteristic of aging in many species [119,139,140]. However, such a decline with age was not observed in the ant *Pheidole dentata*: old workers neither experienced a deterioration of cognitive functions nor changes in the brain characteristic of aging [141]. These results question whether neural deterioration is a hallmark of aging in social insects. Although in our study no tests were performed regarding the decline of cognitive performance and sensory perception in old workers, the higher expression of odorant receptors (four DETs, see S2 Table) and the expression of *idh* involved in the protection against oxidative stress damage in dopaminergic neurons [136], might be a sign of no aging in *C*. *secundus* workers. In contrast, we consider the upregulation with age (HO) of *ref(2)p* in kings and the downregulation of *Ppt1* (HY) in queens as clear signs of nervous system degeneration and aging. *Ref(2)p* is a conserved marker of neuronal aging [100,142], and the deficiency of *Ppt1* in flies is associated with an abnormal accumulation of lipofuscin in the nervous system (lipofuscin accumulates with age) and a reduced lifespan [96]. Some neurodegenerative diseases in humans are caused by mutations in *Ppt1* [143].

### The role of immunity in aging

The loss of immunocompetence and the dysregulation of the immune system are considered a hallmark of aging [144–146], but how aging and immunity influence each other is still not completely understood [147]. In *D*. *melanogaster*, an overexpression of immunity genes is characteristic of aging [148–150]. An immunity response clears pathogens but it can also cause tissue damage via inflammation. Thus a hyper-activation of the immune system can represent a state of immunopathology and promote aging [146]. Overall, all castes in *C*. *secundus* showed an increase in expression of immunity genes with age, but to properly interpret these results the caste-specific interplay between immunity and oxidative stress response should be considered. Immune defense is associated with an upregulation of oxidative stress response genes [151–154]. Hence, the overexpression of immunity genes and the downregulation of stress and oxidative stress response genes in *C*. *secundus* reproductives (but not workers) could reflect the lack of homeostasis between pathways, and be eventually interpreted as a sign of aging.

### Typical aging pathways in *C*. *secundus*

We found few but relevant DETs connected with typical aging pathways like the IIS or the TOR pathway: *Imp-12*, *prmt1* and *cora*, which were all over-expressed in young compared to old queens (*Imp-12* also in young kings). These genes have been described to directly or indirectly interact with *foxo* (IIS pathway). *Imp-l2* binds insulin like peptides (ILPs) and its over-expression in *D*. *melanogaster* leads to a lifespan extension [155,156]. Thus a decline in expression of *Imp-l2* in old *C*. *secundus* reproductives might again be a sign of aging. *Prmt1* encodes an arginine methyltransferase, which, in mammals, methylates *foxo* [157,158]. The function of *cora* is unknown, yet for *D*. *melanogaster*, experimental evidence exists that it physically interacts with *foxo* (IIS pathway) and *rictor* (TOR pathway) [116]. ATPsynD, differentially expressed in kings (HY), was another gene connected to the TOR pathway and involved in aging. The downregulation of this gene in *D*. *melanogaster* conferred a lifespan extension to females fed with a low carbohydrate-to-protein diet [113]. The effect of this gene in the context of termites and their diet should be explored in future studies. The expression patterns of *Imp-l2*, *prmt1*, *ATPsynD* and *cora* in *C*. *secundus* suggest that, as in other insects/animals lifespan determination and aging processes might be modulated by the typical aging pathways IIS and TOR.

## Conclusions

Our results demonstrate that reproductives of the lower termite *C*. *secundus* show signs of senescence. The age related changes in expression suggest that aging might be linked to TE activity, oxidative stress and ‘wear and tear’, and that it may partly be modulated by the IIS pathway, immunity response and epigenetic modifications. For the totipotent workers, which can become reproductives, we did not find evidence of aging but rather a strong signal of metabolism, growth and development as indicated by the identity of DETs and the GO enrichment analysis. Hence our results contrast strongly with those for the higher termite *M*. *bellicosus* where reproductives do not show signs of aging while the major workers did [18]. This is in line with the general prediction of life history theory for organisms with reproductive options that aging should only start after having reached maturity [8]: the totipotent *C*. *secundus* workers are immatures (they are larval instars) that can develop into reproductives and hence should not age. *M*. *bellicosus* workers are also non-adults, but in contrast they are irrevocably sterile and experience much higher extrinsic mortality risks which should both favor faster aging. The degree to which workers can reproduce differs across social insect species and generally correlates with sociality [159]. Therefore, we suggest that aging in reproductives and workers depends, among other factors, on the degree of worker’s reproductive options which should be tested in future studies.

## Supporting information

S1 Fig Shared differentially expressed transcripts (DETs) with age (old vs young) in opposite direction between *C*. *secundus* queens, kings and workers. The heatmap depicts the log2fold changes in expression; each row corresponds to a caste and each column to a transcript. NS: differential expression not significant (padj>0.05).
(EPS)

S1 Table Detailed sample information. (XLSX)

S2 Table Transcript expression differences between old and young individuals for *Cryptotermes secundus* queens, kings, and workers, respectively. (XLSX)

S3 Table DAVID GO enrichment for the differentially expressed transcripts (DETs) of queens, kings, and workers, respectively. (XLSX)

S4 Table Cross species comparison of differentially expressed genes between old and young individuals. *Drosophila melanogaster* one-to-one orthologs and homologs were used for the comparisons.
(XLSX)

S5 Table Full results of cross species comparison of GO enriched terms. (XLSX)

S6 Table Comparison of *Cryptotermes secundus* differentially expressed transcripts (DETs) with candidate aging markers known from *Drosophila melanogaster*. (XLSX)

S7 Table Transposable element (TE) related DETs and Dfam results. (XLSX)
